# miR-Q: a novel quantitative RT-PCR approach for the expression profiling of small RNA molecules such as miRNAs in a complex sample

**DOI:** 10.1186/1471-2199-9-34

**Published:** 2008-04-10

**Authors:** Soroush Sharbati-Tehrani, Barbara Kutz-Lohroff, Ramona Bergbauer, Jutta Scholven, Ralf Einspanier

**Affiliations:** 1 Institute of Veterinary-Biochemistry, Freie Universität Berlin, Oertzenweg 19 b, 14163 Berlin, Germany

## Abstract

**Background:**

MicroRNAs (miRNAs) are small endogenous non-coding interfering RNA molecules regarded as major regulators in eukaryotic gene expression. Different methods are employed for miRNA expression profiling. For a better understanding of their role in essential biological processes, convenient methods for differential miRNA expression analysis are required.

**Results:**

Here, we present the miR-Q assay as a highly sensitive quantitative reverse transcription PCR (qRT-PCR) for expression analysis of small RNAs such as miRNA molecules. It shows a high dynamic range of 6 to 8 orders of magnitude comprising a sensitivity of up to 0.2 fM miRNA, which corresponds to single copies per cell. There is nearly no cross reaction among closely-related miRNA family members, which points to the high specificity of the assays. Using this approach, we quantified the expression of let-7b in different human cell lines as well as miR-145 and miR-21 expression in porcine intestinal samples.

**Conclusion:**

miR-Q is a cost-effective and highly specific approach, which neither requires the use of fluorochromic probes, nor Locked Nucleic Acid (LNA)-modified oligonucleotides. Moreover, it provides a remarkable increase in specificity and simplified detection of small RNAs.

## Background

RNA interference (RNAi) is an evolutionarily-conserved process that modulates gene expression. Recently, miRNA molecules have been described as playing a major role among non-coding small interfering RNAs. MiRNAs represent an abundant and highly conserved family of endogenous single-stranded small RNA molecules of approximately 20–25 nucleotides in length. The high evolutionary conservation of miRNAs from distantly related species indicates their role in various crucial biological processes. Recent studies have demonstrated that miRNAs act as major regulators of developmental timing, cellular differentiation, apoptosis, and signalling pathways [1]. In plants, RNAi has also been described as a phenomenon called post-transcriptional gene silencing [2]. Within this context, miRNAs posses similar functions in plants such as organ development, signal transduction, and response to environmental stress [3].

Animal miRNAs derive from long endogenous primary transcripts with a local stem-loop structure, which is successively cleaved by cellular RNases to build the mature miRNA [4]. One of the strands interacts with the RNA-induced silencing complex (RISC) and binds to a target site, which is located in the 3' UTR of the related mRNA. Most animal miRNAs bind to their targets with incomplete complementarity [1], while their regulatory impact is mainly based on the reduction of translation efficiency, rather than on enhanced mRNA degradation [5].

Due to the enormous regulative importance of miRNAs, research on miRNA expression analysis has recently increased. Although miRNAs represent a relatively abundant class of transcripts, their expression levels vary greatly among species and tissues [6]. Various methods are employed for detection and differential expression analysis of miRNAs in biological samples. Initial miRNA expression studies were performed by means of Northern blot analysis [7]. This method is well-established but is very laborious and highly limited, regarding sample throughputs. Recently, Microarrays have been used for genome-wide miRNA profiling based on different processing chemistries [8-10]. Microarrays provide a robust platform for fast screening and comparative expression analysis of miRNAs, but are limited, regarding accurate quantification of gene expression and need high quantities of RNA. Thus, less abundant miRNAs often escape detection by technologies such as cloning, Northern blot analysis and Microarray. A single-molecule method for quantification of miRNA expression was introduced by Neely et al. [11]. Although this technique represents a modern alternative, it has a detection limit of 500 fM. Additionally, the method is utilised by a cost-intensive device, which may be unaffordable for many research groups.

A highly convenient and reliable method for differential gene expression analysis is considered to be qRT-PCR [12], which exhibits high sensitivity and target specificity. It is 1000-fold more sensitive than methods that are based on hybridisation [13] and can even detect a few target copies [14]. However, the small size of miRNAs requires substantial modification of this method because ordinary PCR primers are usually the same size as the small RNA molecules being analysed. The first miRNA real-time PCR approach was based on detection and quantification of miRNA precursors [15,16]. However, other studies have shown that the cellular steady-state level of miRNA precursors does not correspond to cellular concentrations of mature miRNAs [17,18]. Chen and colleagues [19] have developed a quantitative stem-loop RT-PCR for detection of mature miRNAs that is based on TaqMan assays. Their assay displays high sensitivity and dynamic range and allows for discrimination between members of miRNA families such as let-7. This assay and a few others have recently become commercialised and are rather costly. Raymond et al. [18] have designed a SYBR Green real-time RT-PCR for detection of mature miRNA molecules using LNA-modified primers. Most of their developed assays are described as being sensitive to femtomolar concentrations. However, the performance and sensitivity of 70% of the analysed assays strictly depend on the use of LNA-modified primers, which increases the costs of this approach.

Due to the rising general interest in miRNA expression and their regulative impact on important biological processes, more sensitive and competitive alternatives applications are in demand. On this account, we have developed a new cost-effective, highly sensitive, and reliable qRT-PCR for quantification of small RNA molecules such as miRNAs. The method called miR-Q is based on primer extension, followed by a novel quantitative PCR (qPCR) approach that is carried out using three DNA-oligonucleotides. The assay exhibits high sensitivity, linearity, and discriminative power without the use of complex fluorochromic probes or LNA-modified oligonucleotides.

## Results

### General assay design

**Figure 1:**
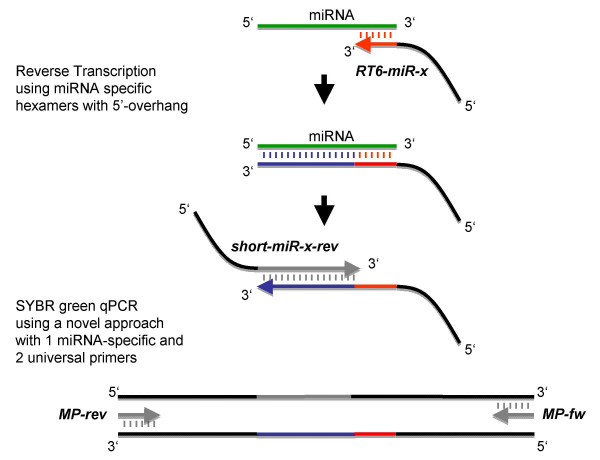
**Schematic description of assay design**. The miRNA is first converted and simultaneously elongated into a cDNA molecule using a miRNA-specific oligonucleotide with 5' overhang (*RT6-miR-x*) and six complementary bases (red). Detection and amplification of the relating cDNA are employed, using a novel PCR approach with three different oligonucleotides at different concentrations within the same assay. The cDNA sequence (blue) is first detected and elongated by a specific oligonucleotide with 5' overhang (*short-miR-x-rev*). Exponential amplification is then performed using two terminal universal primers (*MP-fw *&*MP-rev*).

The novel miR-Q approach has been introduced for detection, quantification, and differential expression profiling of small RNA molecules, in particular miRNAs, in a complex sample. During the first step, miRNA molecules are converted into cDNA and simultaneously elongated by reverse transcription using a miRNA-specific oligonucleotide with 5' overhang (*RT6-miR-x, x *indicates one of the ten particular miRNAs). Subsequently, cDNA molecules are amplified and quantified by means of a novel qPCR approach based on utilising three DNA-oligonucleotides at different concentrations (Figure 1). Primarily, a DNA-oligonucleotide molecule (*RT6-miR-x*) is used for reverse transcription, which comprises two main sequence portions. The terminal six bases at the 3' end of *RT6-miR-x *are miRNA-specific and hybridise to the template RNA molecule of interest. This oligonucleotide molecule also comprises a second sequence portion, enabling the binding of a universal primer (*MP-fw*) to prime the exponential DNA amplification. For subsequent qPCR analysis, another oligonucleotide molecule (*short-miR-x-rev*) is employed comprising a miRNA-specific sequence portion at its 3' end. Therefore, *short-miR-x-rev *can hybridise to the cDNA molecule (complementary to the target miRNA) generated after the reverse transcriptase reaction. In addition, the 5' overhang provides a terminal binding sequence for another universal primer (*MP-rev*). Consequently, two 5' overhangs are introduced to convert and elongate the small RNA molecule. The final exponential amplification is accomplished by means of universal primers (*MP-fw *and *MP-rev*), which hybridise to terminal binding sites of the overhangs. These universal primers are not assay-specific and are therefore employed independently of various analysed miRNA molecules. The universal overhang sequences of the reverse primers as well as RT primers have been deduced from mycobacterial genome, finally being optimised for negligible complementarities and dimer formation. The mentioned sequence parts were used to perform blast searches using the Wublastn option of miRBase, Release 9.2 [20]. These universal overhang sequences show no similarity with any known miRNA using an Evalue cutoff = 20. Even at higher Evalue cutoffs (< 40) only three miRNA molecules show marginal homology to the conserved regions (ebv-miR-BART5, rlcv-miR-rL1-8, and hsa-miR-618).

The final detection and quantification of template miRNA molecules are performed by real-time acquisition of the SYBR Green fluorescence, utilising a calibration curve.

### Assay validation, sensitivity, and dynamic range

Initial assays were performed with miRNA-specific oligonucleotides possessing an overlap of 6 bases with the *RT6-miR-x*, which was used for the initial primer extension reaction to convert and elongate the miRNA molecule in cDNA. Although PCR reactions were performed under stringent conditions, the application of oligonucleotides with 6 overlapping bases produced high background from 30 cycles onwards. Hereafter, we evaluated the use of *short-miR-x-rev *molecules, which featured basically no or, at most, 3 overlapping bases. The use of shortened miRNA-specific reverse oligonucleotides resulted in a higher sensitivity of all assays and a loss of background over 40 cycles.

The dynamic range and sensitivity of all ten miR-Q assays were first proven by performing RT reactions, using the particular synthetic miRNA molecules spiked into 50 ng bacterial total RNA, representing a complex RNA background. Primarily, the assay-specific T_anopt _was evaluated by performing a conventional gradient PCR (annealing temperature gradient: 53–65°C), using an RT reaction prepared with 10 pM synthetic miRNA, with each of the ten containing 50 ng bacterial total RNA as a template. The resulting specific T_anopt _of every assay is indicated in Table 1.

**Table 1:** Oligonucleotides used in this study. The assay-specific annealing temperatures are given in the bottom right column. Differing bases among members of the let-7 family are indicated in red. Binding sequences for universal primers are underlined and the miRNA specific sequences are indicated in bold.

Synthetic miRNA molecules and DNA-oligonucleotides for RT and qPCR	Sequence (5' to 3')	T_anopt _of miR-Q assays [°C]
*let-7a*	uga ggu agu agg uug uau agu u	
*let-7b*	uga ggu agu agg uug ugu ggu u	
*let-7c*	uga ggu agu agg uug uau ggu u	
*miR-16*	uag cag cac gua aau auu ggc g	
*miR-21*	uag cuu auc aga cug aug uug a	
*miR-23b*	auc aca uug cca ggg auu acc	
*miR-27a*	uuc aca gug gcu aag uuc cgc	
*miR-141*	uaa cac ugu cug gua aag aug g	
*miR-145*	guc cag uuu ucc cag gaa ucc cuu	
*miR-200c*	uaa uac ugc cgg gua aug aug g	
*RT6-let-7a*	tgt cag gca acc gta ttc acc gtg agt ggt **aac tat**	
*RT6-let-7b*	tgt cag gca acc gta ttc acc gtg agt ggt **aac cac**	
*RT6-let-7c*	tgt cag gca acc gta ttc acc gtg agt ggt **aac cat**	
*RT6- miR-16*	tgt cag gca acc gta ttc acc gtg agt ggt **cgc caa**	
*RT6- miR-21*	tgt cag gca acc gta ttc acc gtg agt ggt **tca aca**	
*RT6- miR-23b*	tgt cag gca acc gta ttc acc gtg agt gct **ggt aat**	
*RT6- miR-27a*	tgt cag gca acc gta ttc acc gtg agt ggt **gcg gaa**	
*RT6- miR-141*	tgt cag gca acc gta ttc acc gtg agt ggt **cca tct**	
*RT6- miR-145*	tgt cag gca acc gta ttc acc gtg agt ggt **aag gga**	
*RT6- miR-200c*	tgt cag gca acc gta ttc acc gtg agt ggt **cca tca**	
*short-let-7a-rev*	cgt cag atg tcc gag tag agg ggg aac ggc g**tg agg tag tag gtt gta ta**	56
*short-let-7b-rev*	cgt cag atg tcc gag tag agg ggg aac ggc g**tg agg tag tag gtt gtg tg**	56
*short-let-7c-rev*	cgt cag atg tcc gag tag agg ggg aac ggc g**tg agg tag tag gtt gta tg**	56
*short-miR-16-rev*	cgt cag atg tcc gag tag agg ggg aac ggc g**ta gca gca cgt aaa ta**	59
*short-miR-21-rev*	cgt cag atg tcc gag tag agg ggg aac ggc g**ta gct tat cag act ga**	59
*short-miR-23b-rev*	cgt cag atg tcc gag tag agg ggg aac ggc g**at cac att gcc agg g**	58
*short-miR-27a-rev*	cgt cag atg tcc gag tag agg ggg aac ggc g**tt cac agt ggc taa g**	57
*short-miR-141-rev*	cgt cag atg tcc gag tag agg ggg aac ggc g**ta aca ctg tct ggt aaa g**	56
*short-miR-145-rev*	cgt cag atg tcc gag tag agg ggg aac ggc g**gt cca gtt ttc cca gga a**	60
*short-miR-200c-rev*	cgt cag atg tcc gag tag agg ggg aac ggc g**ta ata ctg ccg ggt aa**	59
*5S rRNA fw*	gcc cga tct cgt ctg atc t	60
*5S rRNA rev*	agc cta cag cac ccg gta tt	
*MP-fw*	tgt cag gca acc gta ttc acc	
*MP-rev*	cgt cag atg tcc gag tag agg	

**Figure 2:**
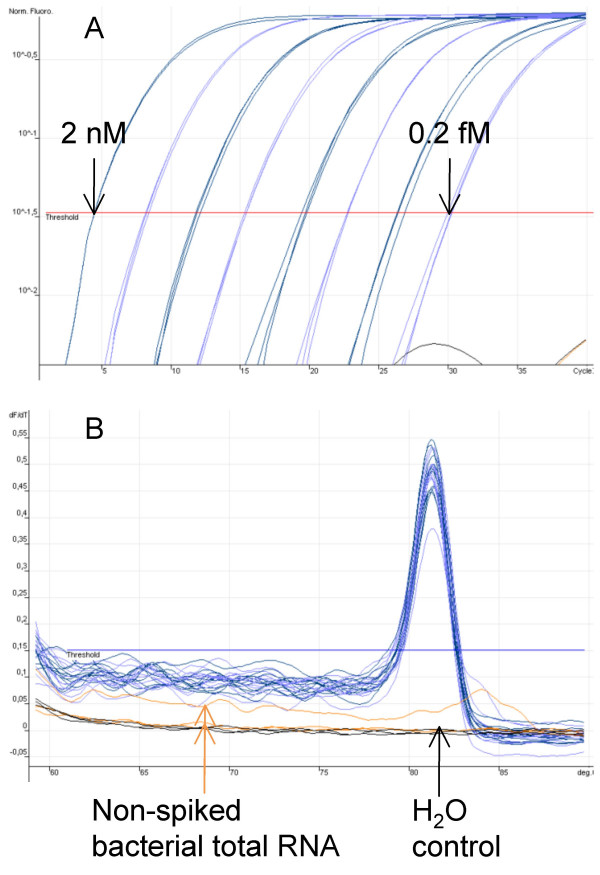
**Amplification plot and melting curve of the proposed miR-145 miR-Q assay**. A) Amplification plot of synthetic miR-145 with 50 ng bacterial total RNA present as complex background. Target input ranged between 2 nM and 0.2 fM in a miR-Q reaction. B) SYBR green melting curve of the miR-145 assay. Neither the non-spiked bacterial total RNA control, nor H_2_O control showed a background signal over 40 cycles.

**Figure 3:**
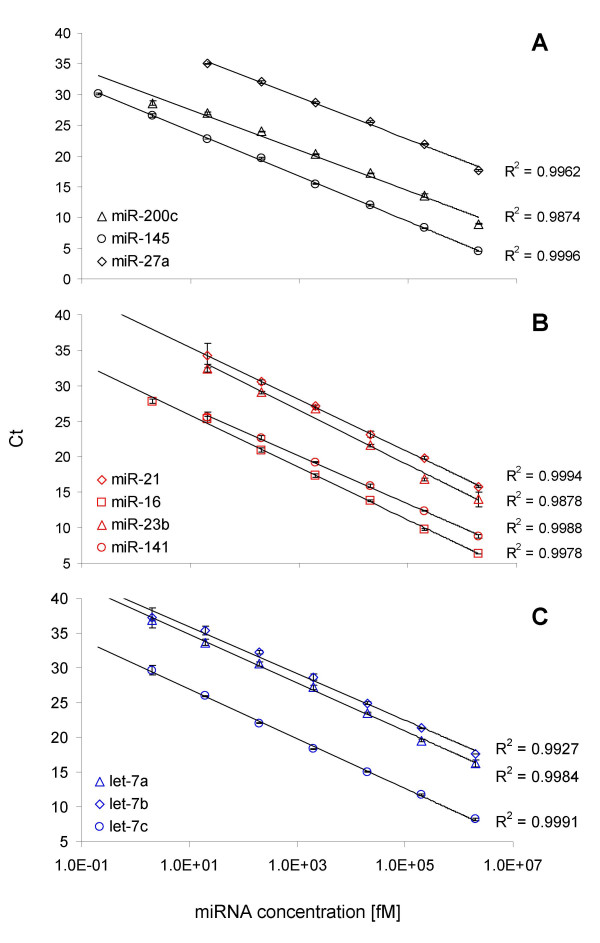
**Dynamic range and sensitivity limit of ten miR-Q assays**. Each value represents the mean (± SD) of three measurements. They showed dynamic ranges between 6 and 8 orders of magnitude, comprising a sensitivity of up to 0.2 fM synthetic miRNA. A) Dynamic ranges of miR-Q assays: miR-200c (black triangles), miR-145 (black circles), and miR-27a (black rhombi). B) Dynamic ranges of miR-Q assays: miR-21 (red rhombi), miR-16 (red squares), miR-23b (red triangles), and miR-141 (red circles). C) Dynamic ranges of miR-Q assays: let-7a (blue triangles), let-7b (blue rhombi), and let-7c (blue circles).

Certain amounts of synthetic miRNAs (10 nM – 1 fM in RT reaction) were applied in order to analyse the dynamic range of the approach. The linearity and sensitivity of the approach were first evaluated using synthetic miR-145, with bacterial total RNA present. The miR-145 assay exhibited excellent linearity over 8 orders of magnitude, detecting as low as 0.2 fM synthetic miR-145 (Figure 2A). The assay exhibited high specificity and sensitivity for miR-145, showing no background signal over 40 cycles either in the non-spiked bacterial total RNA control or in the no template control (Figure 2B). Furthermore, nine distinct miRNA assays were designed and evaluated in the same way using synthetic miRNA molecules and bacterial total RNA as complex background. All of the ten assays showed linearities between the log of miRNA concentration and threshold cycle (Ct) values over more than 6 orders of magnitude. All of the studied assays comprised a high sensitivity showing a minimum detection level of at least 20 fM synthetic miRNA, while most of the assays offered a sensitivity of 2 fM. As indicated in Figure 3, most of the validated assays (miR-200c, miR-16, let-7a, let-7b, and let-7c) showed linearities over 7 orders of magnitude, detecting as low as 2 fM synthetic miRNA. Four of the analysed assays (miR-27a, miR-21, miR23b, and miR-141) showed a dynamic range of 6 logs, while at least 20 fM synthetic miRNA in a miR-Q reaction were measured. All of the calculated coefficients of determination (R^2^) ranged between 0.9874 and 0.9996, which underlined the eminent linearity between Ct values and miRNA input (Figure 3). Negative controls were established for all of the ten studied assays by performing RT reactions with non-spiked bacterial total RNA. All studied miR-Q negative control assays gave neither a detectable, nor specific signal over 40 cycles.

### Assay specificity and cross reaction between let-7 family members

The discriminative power and specificity of the miR-Q approach were analysed for three closely sequence-related members of the let-7 family (let-7a, let-7b, and let-7c), which differed only in one or two nucleotides (Table 1). Each synthetic miRNA molecule was applied to each specific assay and the cross reaction of an assay was determined at different miRNA concentrations by offering unspecific miRNA targets. All experiments were performed along the entire dynamic range of assays (2 nM – 2 fM in a miR-Q reaction). Absolute quantification of assay-unspecific targets was performed using a calibration curve established with specific targets. The percentage of cross reaction values was calculated between assay-specific and unspecific miRNA targets, while the concentration of assay-specific molecules represented the full value. The miR-Q approach showed a high capability to discriminate between miRNA molecules, which differ by less than two nucleotides. Marginal cross reaction was only observed at miRNA concentrations higher than 200 fM synthetic miRNA in a miR-Q assay with minute values ranging from 0% to 0.37%, indicating the high specificity and selectivity of each let-7 assay (Table 2). For miRNA concentrations below 200 fM, only the assay-specific miRNA was detected. The highest cross reaction values (0.37%) were observed using the let-7a assay and synthetic let-7c at high concentrations. No cross reaction at all was detected using the let-7a assay versus let-7b as a target.

**Table 2:** Cross reaction of the miR-Q let-7 assays at different target concentrations. The percentage of cross reaction values was calculated between assay-specific and unspecific miRNA targets, while concentration of assay-specific molecules represented the full value.

	let-7a miR-Q	let-7b miR-Q	let-7c miR-Q
2 nM let-7a	**100**	0.31	0.01
2 nM let-7b	0.00	**100**	0.23
2 nM let-7c	0.37	0.29	**100**
200 pM let-7a	**100**	0.31	0.01
200 pM let-7b	0.00	**100**	0.13
200 pM let-7c	0.24	0.24	**100**
20 pM let-7a	**100**	0.30	0.01
20 pM let-7b	0.00	**100**	0.10
20 pM let-7c	0.24	0.19	**100**
2 pM let-7a	**100**	0.00	0.00
2 pM let-7b	0.00	**100**	0.00
2 pM let-7c	0.00	0.16	**100**
200 fM let-7a	**100**	0.00	0.00
200 fM let-7b	0.00	**100**	0.00
200 fM let-7c	0.00	0.00	**100**
20 fM let-7a	**100**	0.00	0.00
20 fM let-7b	0.00	**100**	0.00
20 fM let-7c	0.00	0.00	**100**
2 fM let-7a	**100**	0.00	0.00
2 fM let-7b	0.00	**100**	0.00
2 fM let-7c	0.00	0.00	**100**

### Comparison of the miR-Q approach with a commercial qRT-PCR detection kit

**Figure 4:**
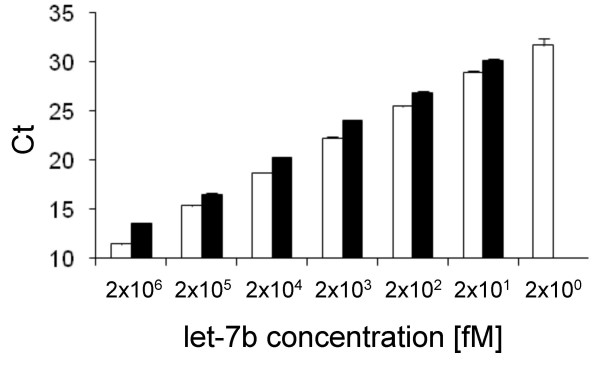
**Detection and quantification of synthetic let-7b in the presence of a complex background using the miR-Q assay and mirVana™ qRT-PCR Detection Kit within the same qPCR run according to the mirVana protocol**. The purchasable let-7b assay showed a sensitivity limit of 20 fM synthetic let-7b (black columns), while the sensitivity limit of the miR-Q assay turned out to be 2 fM synthetic let-7b (white columns). Each column represents the mean (± SD) of three measurements.

Currently, few commercial miRNA qRT-PCR assays are available. The mirVana™ qRT-PCR miRNA Detection Kit for let-7b (Ambion) has been applied to validate the performance of the miR-Q assay. The selected commercial assay is well suited for this purpose, since it is also based on an initial RT reaction, followed by SYBR Green quantification. Since our let-7b assay offered a characteristic medium linearity (7 orders of magnitude), we decided to compare the sensitivity and quantitative power of the miR-Q approach with the competitor using let-7b. Both assays were compared, performing reactions according to the manufacturer's protocol, as well as to our new method. For this purpose, 50 ng of bacterial total RNA was spiked with synthetic let-7b to give final concentrations between 10 nM and 10 fM in RT reaction. cDNAs were quantified in subsequent qPCRs, which were similarly carried out, according to both protocols comparing the performace of both methods within the same qPCR run. Regardless of the two different assay conditions, the miR-Q approach provided a higher sensitivity and linearity, characterised by slightly lower Ct values and a higher dynamic range (Figure 4). While the competitor assay provided linearity over 6 orders of magnitude with a sensitivity limit at 20 fM synthetic let-7b under optimal conditions, as little as 2 fM synthetic let-7b was detected using the miR-Q assay (Figure 4). Both assays showed excellent amplification efficiencies, ranging between 0.95 and 0.98 and R^2 ^> 0.996.

**Figure 5:**
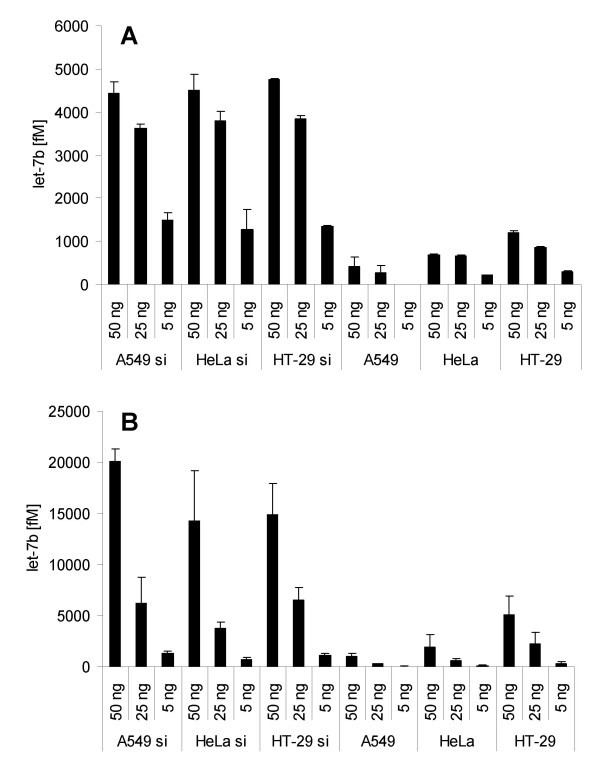
**Side-by-side quantification of let-7b in human total RNA samples isolated from the cell lines: A549, HeLa, and HT-29 in two independent qPCR runs**. Experiments were performed using the miR-Q approach as well as the mirVana™ qRT-PCR Detection Kit. For this purpose, total RNA samples (50 ng/μl) were either spiked with 100 pM synthetic let-7b (A549 si, HeLa si, and HT-29 si) or remained non-spiked (A549, HeLa, and HT-29). RT reactions were performed with 50 ng, 25 ng, and 5 ng of all RNA samples, followed by qPCR detection of let-7b in different runs. Columns represent the mean (± SD) of three measurements. A) let-7b quantification by means of the miR-Q approach using both the spike-in controls and the non-spiked samples. B) Quantification of let-7b by means of mirVana™ qRT-PCR using both the spike-in controls and the non-spiked samples.

Additionally, human total RNA samples from the cell lines A549, HeLa, and HT-29 were employed to validate the performance of the miR-Q assay in real human samples. For this purpose total RNA samples (50 ng/μl) were either spiked with 100 pM synthetic let-7b (spike-in controls) or remained non-spiked (samples). RT reactions were performed with 50 ng, 25 ng, and 5 ng of all RNA samples. The specificity was validated performing side-by-side quantification of let-7b using the miR-Q assay as well as the mirVana™ qRT-PCR miRNA Detection Kit. As shown in Figure 5, let-7b was detected both in spike-in controls and in non-spiked samples using both assays. Both methods revealed consistent high natural let-7b expression in HT-29 cells compared with the other analysed cell lines (Figure 5). It has to be taken into account that the comparative quantification of let-7b by means of the two methods was carried out in two independent qPCR runs. Due to differences between separate qPCR runs, comparative analysis of both methods was performed by calculation of ratios between spike-in controls and non-spiked samples. Both assays exhibited consistent linearity between the spike-in controls and the non-spiked samples demonstrated by the calculated ratio. For example, the miR-Q ratios at different HT-29 RNA concentrations averaged 4.3 ± 0.3 (Figure 5A) and the side-by-side quantification using the mirVana assay resulted in a mean ratio of 3.0 ± 0.2 (Figure 5B).

### Expression of miR-145 and miR-21 in porcine intestinal samples

**Figure 6:**
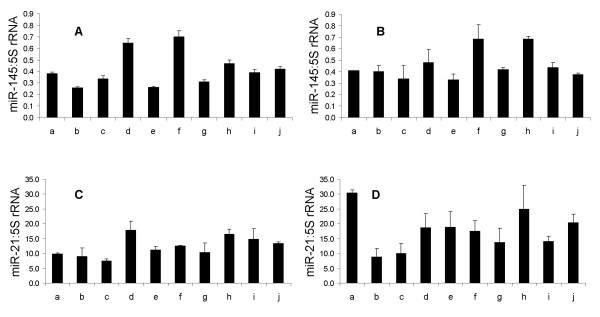
**Expression of miR-145 and miR-21 in porcine intestinal samples**. MiRNA expression values were normalised with relating 5S rRNA expression data and are given as ratios of expression. Each column represents the mean (± SD) of three measurements. A) Expression of miR-145 in ileal samples from ten 31-day-old piglets. B) Expression of miR-145 in jejunal samples from ten 31-day-old piglets. C) Expression of miR-21 in ileal samples from ten 31-day-old piglets. D) Expression of miR-21 in jejunal samples from ten 31-day-old piglets.

In order to test for a demanding practical application, aside from an experimental validation of the assays, the miR-Q approach was used to study differential miRNA expression in biological samples. Therefore, the miR-Q approach was introduced to generate miRNA expression profiles of different porcine intestinal tissues. Target quantity was determined by absolute quantification [12], using a calibration curve established by reverse transcription of serially diluted amounts of the particular synthetic miRNA in the presence of 50 ng bacterial total RNA. The expression profiles of two selected miRNAs (miR-145 and miR-21) were exemplified in 50 ng porcine total RNA isolated from the jejunum or ileum of ten piglets by means of the corresponding miR-Q assays (Figure 6). After measuring 5S ribosomal RNA (rRNA) expression as a housekeeping gene, miRNA data were normalised by calculating the ratios of miRNA and 5S rRNA expression values. The expression of 5S rRNA in porcine jejunum and ileum showed low variation among the ten analysed subjects and turned out to be a reliable housekeeping gene for the normalisation of miRNA values (data not shown). The inter-assay variation of 5S rRNA gene expression among the ten analysed samples was determined as a coefficient of variation (CV) in percent [21]. The calculated CV for the ten ileal samples were 32% and 21% for the jejunal samples, respectively.

Expression of miR-145 and miR-21 in analysed samples was first evaluated by performing initial miRNA-Microarray experiments (data not shown). Subsequent miR-Q analysis demonstrated that both miR-145 and miR-21 were differentially expressed in the jejunal and ileal samples of all ten piglets (Figure 6). There was an obvious individual variance in expression of both miRNAs among the studied subjects. However, miR-145 expression levels in the two different intestinal loci showed similar tendencies. As shown in Figure 6A and 6B, animal f exhibited the highest miR-145 expression among the ten samples, both in jejunum and ileum. Interestingly, both intestinal loci of this animal featured the same miR-145:5S rRNA value, which was twice as high as the lowest expression ratios among the group.

Differential miR-21 expression was also observed among jejunal or ileal samples, respectively. However, there was no distinct concordance of miR-21 expression in the two intestinal loci compared with miR-145 expression. The highest miR-21:5S rRNA expression ratios within the ileal samples were about 2.5 times higher compared with the lowest values, while the highest expression value among the jejunal samples turned out to be 3.5 times higher (Figure 6C and 6D).

Expression values of miR-21 were enhanced in both the ileum and jejunum of all analysed animals compared with miR-145. In ileal samples, a 30-fold higher miR-21 expression value was measured compared with miR-145, while the average values in jejunum were enhanced 40-fold.

## Discussion

The expression of various protein coding genes, their role in development or disease, and their regulation become more and more deciphered. Recently, the small non-coding miRNAs have been highlighted as major modulators of eukaryotic gene expression. It is suggested that up to 30% of human genes may be under the control of miRNAs [22]. MiRNAs are involved in essential developmental processes such as timing, embryogenesis, organogenesis, growth control, and programmed cell death; but they also play a role in human disease, in particular cancer [23]. An increasing number of studies describe aberrant miRNA expression in cancerous tissue. For example, miR-21 was reported as being highly overexpressed in human glioblastoma tumour tissue [24] and in breast tumours, pointing to its function as an oncogene [25]. Other miRNAs such as miR-143 or miR-145 are downregulated in colon carcinomas [26] or in human breast cancer [27]. In many cases, detection of miRNAs and comparative miRNA expression analysis are performed using methods based on probe hybridisation, lacking any amplification step (Northern blot and Microarray). Despite these methods being well established, they are rather limited regarding sensitivity and accurate quantification. Otherwise, due to its high level of sensitivity, accuracy, and practical ease, qRT-PCR is accepted as being a powerful technique in comparative expression analysis in life sciences and medicine [28]. In consideration of the aforementioned facts, quantification of miRNA expression by real-time PCR (e.g. as a diagnostic tool in cancer research) is the method of choice. However, few qRT-PCR approaches are adapted to small-sized miRNAs [18,24] and are currently made available by companies. Our miR-Q approach represents a new, alternative method, which exhibits advanced discriminative power and sensitivity. It is based on primer extension, followed by amplification and quantification of the corresponding cDNA, based on the SYBR Green intercalating chemistry. To establish a novel PCR approach for RNA molecules (< 30 nt) such as miRNAs, three different oligonucleotides were simultaneously employed, one miRNA-specific molecule (*short-miR-x-rev*) for the detection of the corresponding cDNA and two universal primers for the exponential amplification of the product.

We established the described 10 miR-Q assays by randomly choosing miRNAs of interest. Since all of the validated assays proved to be highly sensitive and specific and because of the nature of the overhang sequences, we conclude that these sequence parts can be utilised for ubiquitous detection of small RNA molecule. The linearity and sensitivity of the proposed approach apparently correlates with the length of the detected miRNA molecule. According to miRBase sequence data [20], miR-145 has an extended length of 24 nt, displaying the longest molecule among the miRNAs in this study. Consequently, the most sensitive assay among the validated ten assays (linearity over 8 orders of magnitude) turned out to be the miR-145 assay, featuring an advanced sensitivity limit. Moreover, most of the analysed miRNA molecules with a length of 22 nt exhibited enhanced assay linearity, compared with shorter molecules. Currently, 475 human miRNAs are published in miRBase (Release 9.2). Based on these data, 67% of known human miRNAs are at least 22 nt in length. Accordingly, it can be suggested that the general detection limit of the miR-Q approach would be about 1 fM miRNA. This value may even be underestimated, since 27% of known human miRNAs are longer than 22 nt. Different tissues or cell types may contain diverse amounts of RNA. But according to our calculations, a general detection limit of 2 fM correlates with less than 5 measured copies of miRNA molecules per cell. Hence, the proposed approach offers a cost-effective alternative, exhibiting improved sensitivity and specificity compared with other existing methods without using fluorochromic hybridisation probes or LNA-modified oligonucleotides [18,19]. Recently, qRT-PCR protocols for profiling of mature miRNAs have been introduced where the entire miRNA population contained in samples is reverse transcribed at the same time (Invitrogen or Qiagen). The application of specific RT primers, however, provides an increased specificity of the assays and reduces cross reaction between closely related miRNA molecules. The gain of higher sensitivity over existing assays is also underlined by comparison of our let-7b assay with a purchasable kit. While the mirVana™ qRT-PCR miRNA Detection Kit for let-7b provided lower detection limit, the miR-Q let-7b assay turned out to possess an enhanced dynamic range. Furthermore, the miR-Q assay turned out to be highly discriminative, exhibiting almost no cross reaction between related miRNA molecules, which differed by at least one base. As shown in the let-7a assay versus let-7b as a target, the optimisation of the approach's discriminative power can be achieved by positioning the terminal 3' nucleotide of *short-miR-x-rev *at one of the mismatched bases. Since in real human samples the RNA species are much richer, we performed side-by-side quantification of let-7b in different human cell lines using miR-Q and mirVana™ qRT-PCR and comparing the outcomes of the different assays. The miR-Q results such as the ratios between the spike-in controls and the non-spiked samples were verified using the commercial kit, pointing to the specificity of the miR-Q assay to detect mature miRNA molecules.

To minimise possible sample-to-sample variation due to imbalanced initial total RNA input, we chose 5S rRNA to normalise miRNA expression. Based on the small size of 5S rRNA (representing the small RNA fraction in total RNA samples) and its high conservation level among various species, it appears to be an appropriate gene for the normalisation of miRNAs. Vandesompele et al. [21] studied the normalisation of qRT-PCR data by geometric means. Depending on the selected genes, tissues, and samples, they reported average CV values ranging from 15% to 50%. Despite the fact that we only used the 5S rRNA expression values and not a geometric mean, our calculated CV values correlate well with their data for pooled tissues. Intestinal RNA samples used in this study also represent a complex starting material, since the total RNA was isolated from whole tissues.

miR-21 was reported as being overexpressed in various human cancerous tissues, including colon carcinoma [29,30] and represents an antiapoptotic factor [30]. While common miRNA dysregulation in cancer seems to result in a gain of expression [29], some miRNAs such as miR-145 are downregulated in colorectal carcinomas [26,30]. Dysregulation of these miRNAs in human colorectal cancer points to their potential role in cell proliferation and differentiation in intestinal tissues. Variation in porcine miRNA expression among the ten examined piglets may therefore reflect the individual differences in the intestinal development of the animals. The measured miR-145 and miR-21 expression values in porcine intestinal samples should basically rely on detection of mature miRNA molecules. Within this context, Northern blot analysis and qRT-PCR studies have already shown that the cellular steady-state level of miRNA precursors is negligible, compared with the mature miRNA [17,18].

## Conclusion

Despite the existence of commercial miRNA qRT-PCR approaches, there is an increasing need for fast, reliable, and cost-effective assays, which are adaptable for any research group interested in miRNA detection. Our miR-Q approach provides a linearity of up to 8 orders of magnitude detecting as low as 0.2 fM miRNA molecules.

It offers an alternative method for scientists interested in detecting and quantifying miRNA expression in total RNA samples from different species and tissues.

## Methods

### Oligonucleotides and synthetic miRNA molecules

Ten known miRNA molecules were chosen, inter alia, according to preliminary miRNA-Microarray screening experiments using porcine intestinal samples. These miRNA sequences were chosen from the miRBase Sequence database Release 9.2 [20] and oligonucleotides were designed for every assay (Table 1). Synthetic miRNA molecules were used for the validation of assays. All DNA- and RNA-oligonucleotides were synthesised by Metabion AG (Martinsried, Germany).

### Total RNA isolation from samples

Human total RNA from the cervical carcinoma cell line HeLa (ATCC No.: CCL-2), the colon carcinoma cells HT-29 (ATCC No.: HTB-38), and the lung carcinoma cell line A549 (ATCC No.: CCL-185) as well as porcine total RNA from intestinal tissues were prepared using the mirVana miRNA Isolation Kit (Ambion, Darmstadt, Germany), according to the manufacturer's protocol. The porcine intestinal samples included the jejunum and ileum of ten 31-day-old piglets (EUROC × Pietrain).

In order to validate the new assays, bacterial total RNA was employed as an unspecific complex RNA background in reverse transcription (RT) reactions. RNA was isolated from *Escherichia coli *DH5á using the Invisorb Spin Cell RNA Mini Kit (Invitek, Berlin, Germany), according to the manufacturer's protocol.

The RNA quality and quantity of all samples were proven using the Agilent 2100 Bioanalyzer and the RNA Nano Chips (Agilent, Waldbronn, Germany).

### Reverse transcription

The validation of every assay was performed by RT of certain amounts of the corresponding synthetic miRNA with 50 ng bacterial total RNA present, representing a complex RNA background. Furthermore, the human total RNA as well as the porcine intestinal total RNA were used for reverse transcription.

250 fmol of the miRNA-specific DNA-oligonucleotide (*RT6-miR-x*) with 5' overhang and the RevertAid™ M-MuLV Reverse Transcriptase (Fermentas GmbH, St. Leon-Roth, Germany) were employed to transcribe miRNA into cDNA. A mixture of 50 ng human or porcine total RNA or bacterial total RNA spiked with synthetic miRNA and *RT6-miR-x *was first prepared in a 4 μl volume. The mixture was incubated at 70°C for 5 min and chilled on ice. Then, the volume was brought up to 10 μl by adding the RT-Buffer, 1 mM dNTPs, 100 U Reverse Transcriptase and water. The reaction was incubated at 37°C for 5 min followed by 42°C for 60 min. The enzyme was inactivated by heating at 70°C for 10 min. The standard was prepared using 50 ng of bacterial total RNA spiked with different amounts of synthetic miRNA (10 nM – 1 fM in RT reaction) and was applied for validation and for preparation of qPCR calibration curves. 50 ng of non-spiked bacterial total RNA was used as negative control.

### Quantitative PCR

The optimal annealing temperature (T_anopt_) for every miR-Q assay was first proven by performing a conventional PCR with an annealing temperature gradient ranging from 53 to 65°C. The reaction was performed with 4 nM *short-miR-x-rev*, 100 nM *MP-fw*, and 100 nM *MP-rev *using the Immolase DNA Polymerase (Bioline, Luckenwalde, Germany). The reaction was carried out according to the qPCR conditions (see below) in 25 μl final volume using 2 μl of cDNA, which was obtained from RT reaction with 10 pM of the particular synthetic miRNA. The product size ranged from 82 to 85 bp, depending on the miRNA being detected. After the T_anopt _was determined, the cDNA was quantified using the Rotor-Gene 3000 real-time Detection System (Corbett Life Science, Sydney, Australia) as well as the StepOnePlus™ Real-Time PCR System (Applied Biosystems, Darmstadt, Germany). For this purpose, triplicate measurements of 2 μl cDNA were made in 10 μl final reaction volume. SYBR Green qPCR was performed using the SensiMix DNA Kit (Quantace Ltd., Berlin, Germany), 4 nM *short-miR-x-rev*, 100 nM *MP-fw*, and 100 nM *MP-rev*. The amplification was carried out via the first step at 95°C for 10 min, followed by 40 cycles with 15 s at 95°C, 10 s at the particular annealing temperature (Table 1), and 20 s at 72°C. The fluorescence signal was acquired at 72°C and the Ct values were converted into fM miRNA, using a miRNA-specific calibration curve. MiRNA amounts were determined by triplicate measurements for each sample, compared with a calibration curve established by reverse transcription of serially diluted amounts of the particular synthetic miRNA in the presence of 50 ng bacterial total RNA. The RT reaction of non-spiked bacterial total RNA samples and no template controls were used as negative controls.

### 5S ribosomal RNA normalisation

Sample-to-sample variation of miRNA expression in porcine intestinal samples was corrected by normalisation with 5S rRNA chosen as a housekeeping gene. Firstly, 50 ng of porcine total RNA was reverse-transcribed using the RevertAid™ M-MuLV Reverse Transcriptase (Fermentas GmbH), according to the manufacturer's protocol using random Hexamers. 5S rRNA molecules were quantitated by triplicate measurements of 2 μl cDNA in 10 μl final reaction volume. SYBR Green qPCR was performed using the SensiMix DNA Kit (Quantace Ltd.) and 0.2 μM of each primer *5S rRNA-fw *and *5S rRNA-rev *(table 1). Amplification was carried out via the first step at 95°C for 10 min, followed by 40 cycles with 15 s at 95°C, 10 s at 60°C and 20 s at 72°C, providing an amplicon of 114 bp. The fluorescence signal was acquired at 72°C and Ct values were converted into fg 5S rRNA per qPCR reaction using a calibration curve, which was established by serial dilutions of the corresponding PCR product. Normalisation was performed by calculating the miRNA:5S rRNA expression ratios.

## Competing interests

The authors declare that neither financial nor non-financial competing interests exist. A European patent application (no. 07 006 897.8) is pending.

## Authors' contributions

SST conceived of the study, performed experiments and analyses, wrote and edited the manuscript. BKL and RB performed experiments and analyses. JS collected the porcine intestinal samples and contributed to data analysis. RE contributed to the writing of the manuscript and to the assay design. All authors read and approved the final manuscript.
